# Chronic Exposure to Low Doses of Dioxin Promotes Liver Fibrosis Development in the C57BL/6J Diet-Induced Obesity Mouse Model

**DOI:** 10.1289/EHP316

**Published:** 2016-10-07

**Authors:** Caroline Duval, Fatima Teixeira-Clerc, Alix F. Leblanc, Sothea Touch, Claude Emond, Michèle Guerre-Millo, Sophie Lotersztajn, Robert Barouki, Martine Aggerbeck, Xavier Coumoul

**Affiliations:** 1 INSERM UMR (Institut National de la Santé et de la Recherche Médicale Unité Mixte de Recherche)-S1124, Paris, France; 2 Université Paris Descartes, ComUE (Communauté d’universités et d’établissements), Sorbonne Paris Cité, Paris, France; 3 INSERM UMR-S955, Hôpital Henri Mondor, Créteil, France; 4 Université Paris-Est, Créteil, France; 5 INSERM UMR-S1166, Paris, France; 6 Université Pierre et Marie Curie, Paris, France; 7 Department of Environmental and Occupational Health, School of Public Health, Université de Montréal, Montreal, Quebec, Canada; 8 AP-HP (Assistance Publique - Hôpitaux de Paris), Hôpital Necker-Enfants Malades, Paris, France

## Abstract

**Background:**

Exposure to persistent organic pollutants (POPs) has been associated with the progression of chronic liver diseases, yet the contribution of POPs to the development of fibrosis in non-alcoholic fatty liver disease (NAFLD), a condition closely linked to obesity, remains poorly documented.

**Objectives:**

We investigated the effects of subchronic exposure to low doses of the POP 2,3,7,8-tetrachlorodibenzo-*p*-dioxin (TCDD), an aryl hydrocarbon receptor ligand, on NAFLD progression in diet-induced obese C57BL/6J mice.

**Methods:**

Male C57BL/6J mice were fed either a 10% low-fat (LFD) or a 45% high-fat (HFD) purified diet for 14 weeks and TCDD-exposed groups were injected once a week with 5 μg/kg TCDD or the vehicle for the last 6 weeks of the diet.

**Results:**

Liver histology and triglyceride levels showed that exposure of HFD fed mice to TCDD worsened hepatic steatosis, as compared to either HFD alone or LFD plus TCDD and the mRNA levels of key genes of hepatic lipid metabolism were strongly altered in co-treated mice. Further, increased liver collagen staining and serum transaminase levels showed that TCDD induced liver fibrosis in the HFD fed mice. TCDD in LFD fed mice increased the expression of several inflammation and fibrosis marker genes with no additional effect from a HFD.

**Conclusions:**

Exposure to TCDD amplifies the impairment of liver functions observed in mice fed an enriched fat diet as compared to a low fat diet. The results provide new evidence that environmental pollutants promote the development of liver fibrosis in obesity-related NAFLD in C57BL/6J mice.

**Citation:**

Duval C, Teixeira-Clerc F, Leblanc AF, Touch S, Emond C, Guerre-Millo M, Lotersztajn S, Barouki R, Aggerbeck M, Coumoul X. 2017. Chronic exposure to low doses of dioxin promotes liver fibrosis development in the C57BL/6J diet-induced obesity mouse model. Environ Health Perspect 125:428–436; http://dx.doi.org/10.1289/EHP316

## Introduction

Non-alcoholic fatty liver disease (NAFLD) is associated strongly with obesity and has become the most common cause of chronic liver diseases in Western countries due to the increasing prevalence of obesity and comorbidities worldwide (Loomba and Sanyal 2013). NAFLD includes a wide spectrum of hepatic histological abnormalities ranging from benign steatosis to pathological non-alcoholic steatohepatitis (NASH) and its fibrotic complications that can progress to life-threatening liver cirrhosis and hepatocellular carcinoma (Angulo 2002). The progression from simple steatosis to NASH is a key concern as it is not fully understood why up to 30% of the obese patients with steatosis will develop aggressive NASH (Vernon et al. 2011). According to the “two-hit hypothesis” model, the “first hit” (insulin resistance, obesity, genetic factors) causes accumulation of excess triglycerides in the liver and increases the vulnerability of the liver to the “second hit” (oxidative stress, proinflammatory cytokines, adipokines, mitochondrial dysfunction) that triggers hepatic inflammation and fibrogenesis (Marra and Lotersztajn 2013). Although the exact cause of the inflammation is still difficult to pinpoint, recent studies suggest that the accumulation of triglycerides in the liver (“first hit”) might actually prevent further hepatic damage. Instead, the interruption of triglyceride synthesis could be the initiating event for free fatty acid (FA)-mediated lipotoxicity that leads to NASH and fibrosis (Choi and Diehl 2008; Trauner et al. 2010).

Increasing epidemiological evidence suggests that exposure to environmental pollutants could contribute to the progression of chronic liver diseases by accelerating the progression of fibrosis, particularly in NAFLD patients (Marrero et al. 2005; Zein et al. 2011). The populations of both industrialized and developing countries are exposed commonly to numerous organic pollutants present in the air or in food and several accidents, such as at Seveso (Consonni et al. 2008; CDC 1988), have led to high exposure to such molecules. Among these pollutants, the persistent organic pollutants (POPs), characterized by a long half-life, accumulate life-long due to their storage in the adipose tissue and the liver of exposed organisms (La Merrill et al. 2013; Van den Berg et al. 1994). The toxicity of the POPs depends upon several factors, among which are the molecular structures and the mechanisms of action of these compounds.

The POP 2,3,7,8-tetrachlorodibenzo-*p*-dioxin (TCDD) is the most toxic congener of the dioxin family and is also one of the most potent activators of the aryl hydrocarbon receptor (AhR) (Barouki et al. 2012). Upon ligand binding, the AhR transcriptionally activates enzymatic and transport machinery that allows the elimination of xenobiotics through detoxification processes. However, these processes also can lead to toxicity, due to undesirable chemical reactions, such as oxidative stress (Wilson and Safe 1998). It has been proposed that environmental factors trigger the progression of NAFLD to NASH through the enhanced production of reactive oxygen and/or nitrogen species (Begriche et al. 2011; He et al. 2013). In addition to its role in detoxification, the AhR has been found to affect lipid metabolism and to participate in the development of hepatic steatosis. In rodents, TCDD induces fatty liver via an AhR-dependent mechanism increasing free FA uptake while inhibiting FA β-oxidation, *de novo* lipogenesis and very low-density lipoprotein (VLDL) secretion (Angrish et al. 2012; Lee et al. 2010). Furthermore, our own work (Pierre et al. 2014) and that of others (He et al. 2013) have shown that exposure to a high dose of TCDD leads to hepatic inflammation and liver fibrosis in mice.

Our aim was to investigate the effect of subchronic exposure to a low dose of TCDD on NAFLD progression in the C57BL/6J mouse diet-induced obesity experimental model. We hypothesized that an exposure to 5 μg/kg of TCDD for 6 weeks, combined with the consumption of a moderately high- fat diet (HFD; 45% energy from fat) for 14 weeks, may alter hepatic lipid metabolism and increase inflammation that could aggravate the steatosis that arises following either treatment alone and promote the development of fibrosis in the obese mice.

## Methods

### Animal Experiments

Mice were housed in temperature- and humidity-controlled rooms, kept on a 12-hr light-dark cycle, and provided unrestricted amounts of food and water. Body weight and food intake were monitored weekly throughout the experiment. The animal treatment protocol was approved by the bioethics committee of the Paris Descartes University (authorization no. CEEA34.MA.003.12.) and all of the animals received humane care in accordance with the Guide for the Care and the Use of Laboratory Animals (NRC 2011).

Upon arrival, 60 male C57BL/6J mice (Janvier Laboratories) of 7 weeks of age (about 22 g body weight) were fed a purified low-fat diet (LFD; 10% energy from fat) (D12450B, Research Diets, Brogaarden). After 1 week of acclimatization, the mice were divided into two weight-matched groups (*n* = 30). One group was maintained on the LFD whereas the other one was switched to a HFD (D12451, Research Diets), which contained 45% energy from fat, for 14 weeks. During the last 6 weeks of the diet intervention, the mice from each group were injected intra-peritoneally (200 μL/25 g) once a week with either 5 μg/kg TCDD (LGC Standards) diluted in corn oil (Sigma) (*n* = 16) or the vehicle (nonane diluted in corn oil, Sigma) (*n* = 14). C57BL/6J mice display high inter-individual variability characterized by the presence of low and high weight gain individuals (Koza et al. 2006), that could impact their liver functions, particularly under HFD (Duval et al. 2010). Therefore, on the basis of the leptin and body weight gain measures at week 5, potential low and high weight gain individuals in the LFD and HFD groups were equally distributed into the sub-groups destined for treatment or not with TCDD in order to avoid a biased TCDD effect (see Table S1 and Figure S1). At week 5 and week 13, a few drops of blood were collected as described below, after food was removed between 0800 and 1400 hours to allow the consistent determination of metabolic parameters (referred to as “fasted” measurements in the text). Five days after the last injection, *ad libitum* fed mice were anesthetized with isoflurane and blood was drawn through retro-orbital sinus puncture prior to sacrifice of the mice by decapitation. The liver and white adipose tissues (epididymal and inguinal) were removed, weighed, and either snap-frozen in liquid nitrogen or, for histology, fixed in buffered formalin and processed for paraffin embedding. Serum and plasma samples were obtained after centrifugation of the blood. All samples were stored at –80°C until use.

At the end of the experiment, two mice (from the LFD subgroups) displayed abnormalities (ex: immobility, tremors) and were excluded from the analyses [final group sizes: LFD-fed mice (LF-ctrl, *n* = 13); LFD-fed mice exposed to TCDD (LF-tcdd, *n* = 15); HFD-fed mice (HF-ctrl, *n* = 14); and HFD-fed mice exposed to TCDD, (HF-tcdd, *n* = 16)].

### Blood Measurements

Blood glucose levels were determined using a glucose meter (Accu-Chek performa, Roche). Serum aspartate aminotransferase and alanine aminotransferase activities were measured on an automated analyzer in the Biochemistry Department of the Henri Mondor Hospital. Plasma leptin and insulin levels were quantified by ELISA (R&D Systems and Alpco, Eurobio Laboratories, respectively).

### Quantification of Triglycerides

Lipids were extracted with acetone from 80 mg of liver using a TissueLyser LT (Qiagen) and triglycerides were determined enzymatically (DiaSys), as previously described (Louvet et al. 2011).

### RNA Extraction and Quantitative Real-Time PCR (qPCR)

Total RNA was isolated from the liver with TRIzol reagent (Invitrogen) and purified using the RNeasy minikit (Qiagen), according to the manufacturer’s instructions. RNA reverse-transcription and qPCR were performed as described in Pierre et al. (2014). PCR primer sequences (see Table S2) were ordered from Eurogentec. The relative mRNA levels were estimated using the delta-delta Ct method with the geometric mean of *Gapdh*, *Ppia/cyclophilin* and *Hprt* as the reference.

### Histology

Liver paraffin sections (5 μm) were stained with hematoxylin-eosin or picro-sirius red by standard procedures. Slides were examined by brightfield microscopy. Picro-sirius red stained areas from two fields (200× magnification) per mouse were quantified with ImageJ software (http://imagej.net/Downloads).

### Statistical Analyses

The results are expressed as the mean ± standard error of the mean (SEM) and were analyzed by the Kruskal-Wallis test of the agricolae pack in the R software (version 3.0; R Project for Statistical Computing). A *p*-value < 0.05 was considered to be significant.

## Results

### TCDD Dose to Combine with the Diet Intervention

In a preliminary part of the study (see “Supplemental Results: Determination of the threshold dose of TCDD that induces liver fibrosis” in the Supplemental Material), we first established, using dose-response experiments (see Figure S2), that the threshold subchronic dose of TCDD, which induces liver fibrosis in the mice, was between 1 and 10 μg/kg TCDD. We thus chose to use 5 μg/kg TCDD in the following experiments. With a physiologically based pharmacokinetic model, the intra-peritoneal injection of 5 μg/kg TCDD is predicted to give a final concentration of TCDD in the serum of mice that is below 70 ppt, which is coherent with values for highly exposed human populations (see Figure S3).

### TCDD Does Not Affect HFD-Induced Obesity

**Figure 1:**
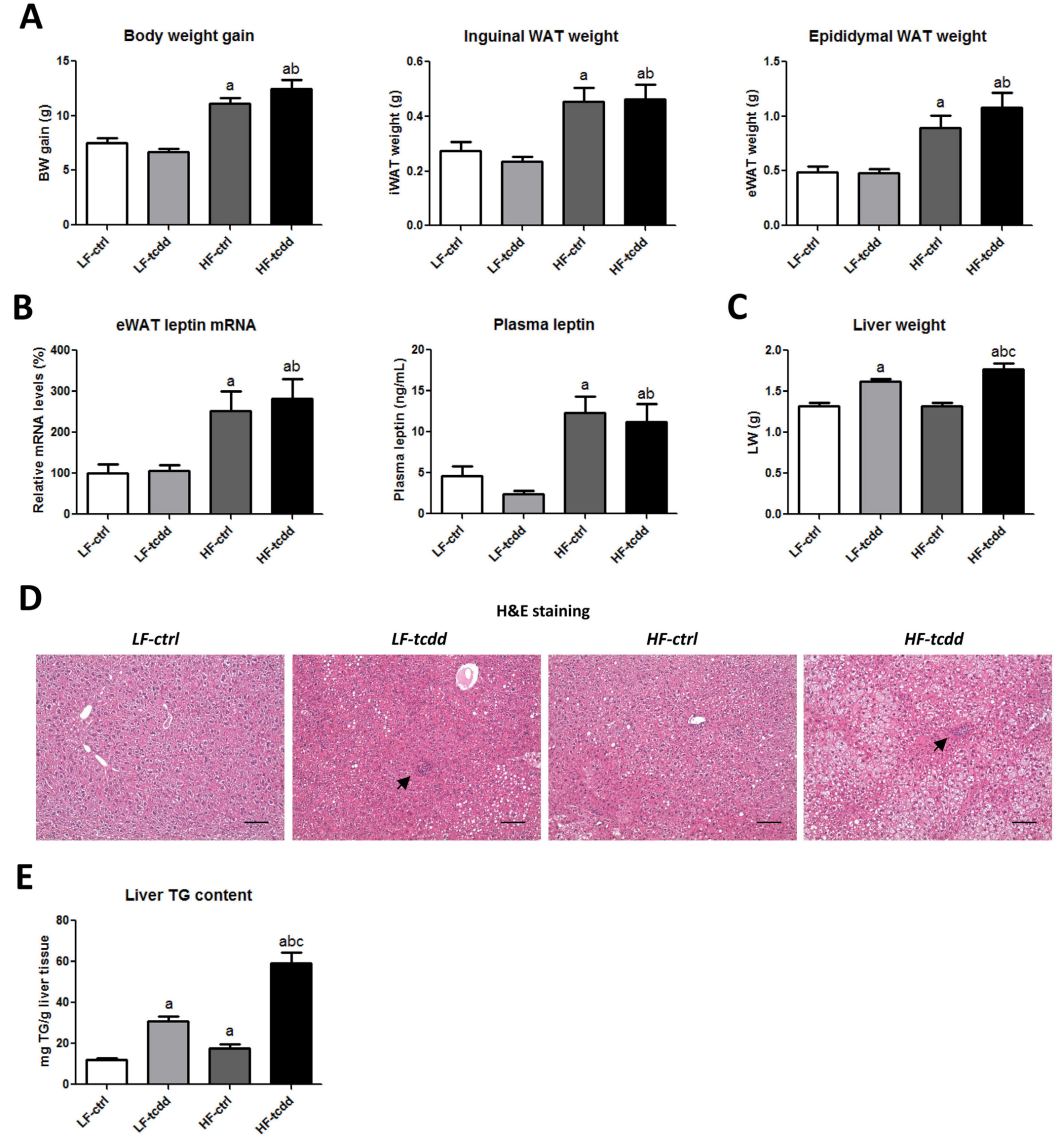
Effect of TCDD on HFD-induced obesity and hepatic steatosis. Mice fed either a LFD or a HFD for a total of 14 weeks were injected with 5 μg/kg of TCDD (LF-tcdd and HF-tcdd, respectively) or the vehicle (LF-ctrl and HF-ctrl, respectively) during the last 6 weeks. (*A*) Body weight (BW) gain, inguinal and epididymal white adipose tissue (WAT) weight. (*B*) Leptin mRNA levels in epididymal WAT (eWAT) measured at 14 weeks and plasma fasted-leptin concentrations at 13 weeks. (*C*) Liver weight. (*D*) Hematoxylin-eosin staining (H&E) of representative liver sections of the different groups, black arrows indicate the islets of infiltrated inflammatory cells (bar = 150 μm). (*E*) Hepatic triglyceride content measured at 14 weeks. Note: Data are expressed as mean ± SEM; a, versus LF-ctrl; b, versus LF-tcdd; c, versus HF-ctrl; *p* < 0.05.

To test the hypothesis that subchronic exposure to low doses of AhR ligands could be a cofactor in the development of fibrosis in obesity related-NAFLD, male mice were fed either a LFD or a HFD for 14 weeks and were injected weekly with either 5 μg/kg TCDD (LF-tcdd and HF-tcdd, respectively) or the vehicle (LF-ctrl and HF-ctrl, respectively) for the last 6 weeks of the diet intervention. After 14 weeks of diet intervention, the four groups of mice had received an isocaloric energy intake as based on the estimate of food intake (data not shown). The HFD led to significant increases in body weight, in inguinal and epididymal white adipose tissue weight, as well as in epididymal white adipose tissue leptin mRNA levels and plasma fasted-leptin concentrations in the mice, as compared to the LFD, with no difference between TCDD-treated and control groups (Figure 1A,B). Fasted glycemia and fasted insulinemia were not altered significantly by the experimental protocol (data not shown). These results confirm that the HFD intervention induced the first signs of obesity and that TCDD had no significant effect on these obesity-related parameters.

### TCDD Worsens HFD-Induced Hepatic Steatosis

In contrast, subchronic exposure to TCDD was associated with a moderate but significant increase in liver weight, whatever the diet (Figure 1C). Hematoxylin-eosin staining of liver sections (Figure 1D) showed that, in control mice, the HFD led to steatosis with no sign of inflammation as compared to LF-ctrl mice, which displayed a normal liver histology. In contrast, TCDD injections in LFD mice led to steatosis together with the infiltration of inflammatory cells grouped in islets. Strikingly, in HFD mice, TCDD dramatically worsened the steatosis, which was accompanied by the infiltration of inflammatory cells. The quantification of the hepatic triglyceride content demonstrated a cumulative effect of HFD and TCDD on lipid accumulation in the liver (4.9-fold increase), as compared to each parameter alone (1.4-fold or 2.5-fold increases for HFD or TCDD, respectively) (Figure 1E). These observations suggest that a moderate HFD combined with a subchronic exposure to TCDD leads to a worsening of a NAFL-like phenotype towards NASH.

### TCDD Impairs HFD Adaptative Molecular Mechanisms

**Figure 2:**
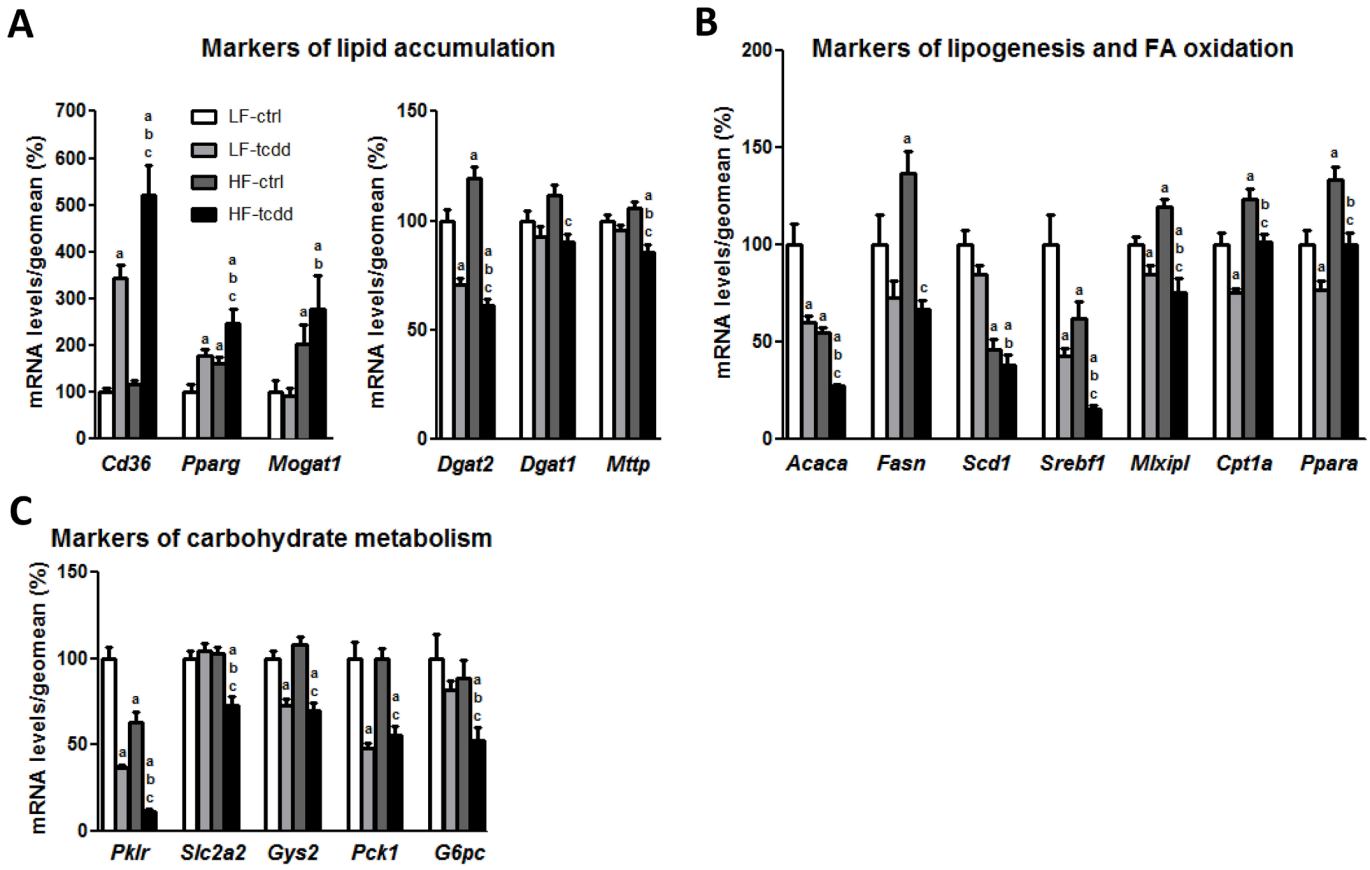
Effect of the co-exposure to TCDD and HFD on the hepatic mRNA levels of markers of lipid and carbohydrate metabolism. Mice fed either a LFD or a HFD for a total of 14 weeks were injected with 5 μg/kg of TCDD (LF-tcdd and HF-tcdd, respectively) or the vehicle (LF-ctrl and HF-ctrl, respectively) during the last 6 weeks. The mRNA levels of hepatic genes were measured by qPCR. Mean expression in the LF-ctrl group is set at 100%. (*A*) Markers of lipid accumulation. (*B*) Markers of lipogenesis and FA oxidation. (*C*) Markers of carbohydrate metabolism. Note: Data are expressed as mean ± SEM; a, versus LF-ctrl; b, versus LF-tcdd; c, versus HF-ctrl; *p* < 0.05.

To decipher the mechanisms involved in the aggravation of steatosis in HF-tcdd mice, the hepatic levels of mRNAs of several genes that are markers of lipid metabolism were quantified by qPCR (Figure 2). HF-tcdd mice exhibit further increases in the expression of the fatty acid transporter *Cd36* and of the nuclear receptor *Pparg*, involved in lipid storage, as compared to LF-tcdd or HF-ctrl mice. Moreover, the expression of *Mttp*, a crucial enzyme for very low-density lipoprotein secretion, was significantly decreased in the HF-tcdd mice although there was no effect in HF-ctrl or LF-tcdd mice compared to LF-ctrl animals (Figure 2A). However, exposure to TCDD counteracted the HFD-induced increase in expression of *Dgat2*, a major enzyme of triglyceride synthesis and the co-treatment also decreased the expression of *Dgat1*, whereas either treatment alone had no effect on this gene. In contrast, the expression of *Mogat1*, the upstream enzyme of the monoacylglycerol pathway, was increased by HFD, without any additional effect of TCDD treatment (Figure 2A). These results suggest an inhibition of triglyceride synthesis in HF-tcdd mice, potentially due to an adaptive feedback mechanism related to triglyceride accumulation. Furthermore, TCDD, together with the HFD, further decreased the expression of both *Srebf1/Srebp1c*, a central regulator of lipogenesis, and its target gene, *Acaca*, the rate-limiting enzyme of *de novo* FA synthesis as compared to LF-tcdd and HF-ctrl mice. In addition, TCDD exposure counteracted the HFD-induced increase in expression of *Mlxipl/Chrebp*, another major transcription factor involved in lipid synthesis and its target gene, *Fasn*, an enzyme crucial for palmitate synthesis. In contrast, the expression of the stearoyl coA desaturase-1, *Scd1*, was decreased only by the HFD, with no effect of TCDD (Figure 2B). Moreover, the expression of *Ppara*, a nuclear receptor regulating FA catabolism, and its target gene *Cpt1a*, the rate-limiting enzyme of mitochondrial β-oxidation, were decreased in HF-tcdd mice as compared to HF-ctrl mice (Figure 2B). This suggests that TCDD prevented a physiological adaptative up-regulation of FA catabolism in HFD mice. Finally, the expression of several genes involved in carbohydrate metabolism, such as the key enzyme of glycolysis *Pklr* and the hepatic glucose transporter *Slc2a2/Glut2* were diminished in HF-tcdd mice (Figure 2C), which indicates additional disruption of energy homeostasis. Together, these results suggest that exposure to TCDD worsens the effects of the HFD by counteracting the adaptative mechanisms triggered by the diet. This is coherent with the dramatic increase in the accumulation of triglycerides in the liver.

### TCDD Promotes Liver Fibrosis Development in Obese Mice

**Figure 3:**
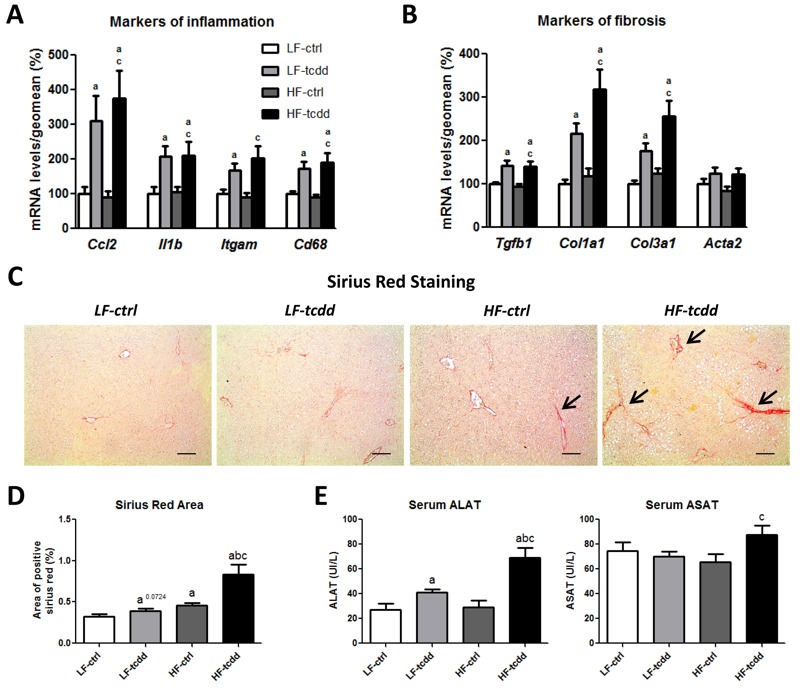
Effect of the combined exposure to TCDD and HFD on the development of hepatic fibrosis. Mice fed either a LFD or a HFD for a total of 14 weeks were injected with 5 μg/kg of TCDD (LF-tcdd and HF-tcdd, respectively) or the vehicle (LF-ctrl and HF-ctrl, respectively) during the last 6 weeks. The hepatic mRNA levels of markers of (*A*) inflammation and (*B*) fibrosis were measured by qPCR. Mean expression in the LF-ctrl group is set at 100%. (*C*) Picro-sirius red staining shows fibrotic scars of collagen I and III (large black arrows, bar = 150 μm). (*D*) Quantification of picro-sirius red staining. (*E*) Serum alanine (ALAT) and aspartate (ASAT) aminotransferase activities. Note: Data are expressed as mean ± SEM; a, versus LF-ctrl; b, versus LF-tcdd; c, versus HF-ctrl; *p *< 0.05.

To further characterize the impairment of liver function in HF-tcdd mice, the levels of the mRNAs of genes involved in inflammation and fibrosis were analyzed by qPCR. TCDD induced the gene expression of inflammatory markers, such as the chemokine *Ccl2/Mcp1*, the interleukin *Il1b* and the macrophage *Itgam/Cd11b* integrin and *Cd68* glycoprotein (Figure 3A), to the same extent in the LF-tcdd and HF-tcdd groups, consistent with the histological observations (Figure 1E). Similarly, the mRNA levels of fibrotic markers, such as the pro-fibrogenic cytokine *Tgfb1*, and the two major collagen fiber components *Col1a1* and *Col3a1*, were increased by the treatment with TCDD independently of the diet and there was no modification in the level of *Acta2/aSma* mRNA among the four groups of mice (Figure 3B). However, picro-sirius red staining of liver sections showed that fibrosis (assessed as the percentage of picro-sirius red stained areas) was significantly greater in HF-tcdd mice than in LF-tcdd or HF-ctrl mice (Figure 3C,D). This was associated with an increase in the alanine and aspartate aminotransferase activities in HF-tcdd mice, which reflects liver injury (Figure 3E). These results suggest that subchronic exposure to low doses of TCDD is sufficient to increase the number of liver fibrotic scars in obese mice.

## Discussion

Epidemiological studies suggest that there is an increased risk of liver pathologies when individuals are exposed to POPs (Cave et al. 2010; Consonni et al. 2008; Yi et al. 2014; Yu et al. 1997). In particular, an increased incidence of liver cirrhosis has been reported in individuals from the Seveso population and the Korean Vietnam Veterans cohort, who were highly exposed to TCDD (CDC 1988; Consonni et a. 2008; Yi et al. 2014). Even though the prevalence of NAFLD is increasing worldwide along with obesity (Loomba and Sanyal 2013), there are very few studies that have examined the association between exposure to POPs and the development of chronic liver diseases in obese NAFLD patients (Marrero et al. 2005; Zein et al. 2011). However, these studies have focused on AhR ligands (cigarette smoke), other than TCDD. The present study provides evidence that TCDD acts as a cofactor for liver fibrosis progression in a background of obesity in mice.

The preliminary objective of our study was to define experimental conditions to study the combination of TCDD treatment and an obesogenic diet in mouse liver. We performed dose-response experiments to establish that 5 μg/kg TCDD is the threshold dose that leads, after repeated exposure during 6 weeks, to the first signs of liver impairment in C57BL/6J mice. Physiologically based pharmacokinetic modeling of a subchronic exposure of mice to 5 μg/kg TCDD predicted final concentrations of 67 ppt TCDD in blood and 57,000 ppt in liver (wet weight), which are consistent with the concentrations previously described in mouse liver (Boverhof et al. 2005; Vezina et al. 2004). Even if caution must be applied when extrapolating results from mice to humans due to species differences, the concentrations predicted by the model in the blood of our mice (11,551 ppt lipid adjusted) are within the same range as those measured in the blood of the population close to the Seveso industrial accident (zone A, range 15–56,000 ppt) (Eskenazi et al. 2004) or of the Ranch Hand cohort of U.S. veterans exposed to the herbicide Agent Orange (range 318–40,376 ppt) at the time of discharge from Vietnam (Emond et al. 2005). These concentrations range far above the background TCDD levels found in the general population. A large study of dioxin blood levels in the United States [the University of Michigan Dioxin Exposure Studies (https://sph.umich.edu/dioxin/)] reported average TEQ blood levels of 23.9 ppt (lipid adjusted) in adults who were 18 years old or older. In contrast to blood, the concentration of TCDD has been measured only rarely in human liver (Leung et al. 1990) due to a limited access to biopsies. A physiologically based pharmacokinetic model of the Ranch Hand cohort predicts TCDD levels of 5,535 ppt in liver for individuals with 162 ppt TCDD in the blood (Emond et al. 2005). These concentrations suggest that, for highly exposed Seveso residents or U.S. veterans with TCDD concentrations above 40,000 ppt in their blood, their level of TCDD in the liver might be within the same range or even higher than the ones predicted in our rodent model (1,360,000 ppt lipid adjusted). Considering that the C57BL/6J mouse model of diet-induced obesity allows a physiological approach to study the metabolic syndrome and related disorders, such as NAFLD (Duval et al. 2010; Larter and Yeh 2008), we combined the subchronic administration of the threshold dose of TCDD (5 μg/kg) for fibrosis with a 14-week HFD nutritional intervention.

We found that 14 weeks of HFD (45% energy from fat) induced early stages of obesity and subchronic exposure to TCDD did not influence the obese phenotype in contrast to exposure to high doses of TCDD that have been related to cachexia (wasting syndrome) (Kelling et al. 1985). The dose of 5 μg/kg used in this study impaired neither weight gain nor leptin levels (mRNA and hormone) in the LFD and HFD groups. On the contrary, the combination of exposure to TCDD and HFD led to the striking impairment of several liver functions, as shown by the drastic increase in the amount of steatosis, and the accumulation of fibrotic scars.

Gene expression analysis suggested that the exposure to TCDD interfered with the metabolic adaptation to the HFD. Whereas exposure to either HFD or TCDD, alone, is known to induce the accumulation of lipid through distinct alterations of lipid and carbohydrate metabolism (Angrish et al. 2012; Duval et al. 2010; Lee et al. 2010; Patsouris et al. 2006), their combination led to a unique gene expression signature. For example, the addition of TCDD to HFD altered the expression of key genes of lipid metabolism such as *Ppara*, *Mlxipl/Chrebp*, *Cpt1a*, *Fasn* and *Dgat2* in a direction that is opposite to that exerted by HFD alone (decrease instead of increase) whereas TCDD exerts its effects in the same direction as HFD on *Pparg and Cd36* (increase) or *Srebf1/Srebp1c, Acaca* and *Pklr* (decrease). In contrast, exposure to TCDD did not alter the HFD-induced modifications of the expression of *Scd1* (decrease) and *Mogat1* (increase). In addition, only HFD and exposure to TCDD in combination decreased the expression of *Dgat1*, *Mttp*, *Slc2a2/Glut2* as compared to the three other conditions.

Our results suggest that the molecular mechanisms that explain the effects of TCDD and HFD on lipid metabolism are complex and could implicate *i*) a direct regulation of AhR target genes such as Cd36 (Lee et al. 2010) and *ii*) an interference between the AhR and other signaling pathways. Indeed, the addition of TCDD to the HFD impacted the expression level of crucial regulators of lipid and carbohydrate metabolism during an HFD metabolic adaptation. For example, TCDD down-regulates *Ppara* and up-regulates *Pparg*, which is consistent with the compensatory effects described by Patsouris et al. (2006) in PPARa KO mice receiving a HFD. This is consistent also with an interaction of the AhR with the PPARa signaling pathway, as it has been proposed (Lee et al. 2010; Shaban et al. 2004; Wang et al. 2011). Moreover, our moderate HFD intervention increases *Mlxipl/Chrebp* and decreases *Srebf1/Srebp1c* mRNA levels. These results are in agreement with those of a study by Benhamed et al. (2012) that showed that transgenic overexpression of *Chrebp* was associated with a decrease of *Srebp1c* and a “good steatosis” profile. The levels of *Chrebp* and *Srebp1c* mRNAs are decreased by TCDD, even when mice receive a HFD. A physical interaction has been described between SREBP1c and the AhR that leads to the disruption of SREBP1c signaling after AhR activation (Cui et al. 2011). This interaction could be the underlying mechanism that explains the drastic decrease of *Srebp1c* in mice exposed to TCDD. Finally, the down-regulations of both *Ppara* and *Srebp1c* also are consistent with a disruption of both carbohydrate and lipid metabolism and may be at the origin, partially, of the unique profile associated with HFD and TCDD (“bad steatosis”).

The effect of TCDD on the expression of *Cd36*, *Cpt1a*, *Acaca*, *Fasn* and *Pklr* is in accordance with the literature (Angrish et al. 2012; Boverhof et al. 2005; Lee et al. 2010; Sato et al. 2008) and the lack of effect of TCDD on *Mogat1* and *Scd1* mRNA expression in our diet experiment is probably due to the lower dose of TCDD that we used as compared to those found in the literature (Angrish et al. 2011, 2012). Cd36 is a direct transcriptional target of the AhR and the PPARg receptors. TCDD increases Cd36 mRNA expression but this effect is potentiated by the high-fat diet. This might be due to the simultaneous stimulation of the PPARg by TCDD and the HFD, whereas the increase in Pparg mRNA expression after 14 weeks of HFD is not sufficient to induce Cd36 by itself. Interactions between the AhR and the PPAR family might also explain the decreased expression of Cpt1a. Indeed, the AhR interacts negatively with PPARalpha that stimulates the expression of Cpt1a. Similarly, Srebp1c and Chrebp signaling are counteracted by the AhR, and this could explain the expression profiles of their target genes that are involved in fatty acid synthesis such as Fas or Acaca.

However, our results are surprising for *Dgat2* (down-regulation). Angrish et al. (2012), who used an acute exposure to a high dose of TCDD in young mice, reported that *Dgat2* was induced by TCDD. This result, together with the increase in expression of *Mogat1/2*, *Cd36*, and *Fabp* and the decrease in very low-density lipoprotein secretion, was suggested to explain the accumulation of triglycerides. Nevertheless, in a human hepatic cell line, HepaRG, treated with TCDD, we found a similar decrease of *Dgat2* mRNA (Ambolet-Camoit et al. 2015) as in the present work. Although the regulation of this gene is poorly characterized (Postic and Girard 2008), the knock-down of *Dgat2* has been associated with both an improvement of steatosis (Shi and Cheng 2009) and an aggravation of hepatic lesions (Yamaguchi et al. 2007). The down-regulation of *Dgat2* that we observe in the TCDD-injected mice, whatever the diet, might, therefore, indicate poorly regulated triglyceride storage and lipotoxicity. The decrease of Dgat1 in the HF-tcdd group compared to the HF-ctrl group, although modest, indicates that this isoform does not compensate for the decrease in Dgat2 gene expression. Finally, *Mogat1*, which is markedly induced by the combined exposure, might also possess a DGAT activity and could have a compensatory activity (Shi and Cheng 2009).

**Figure 4:**
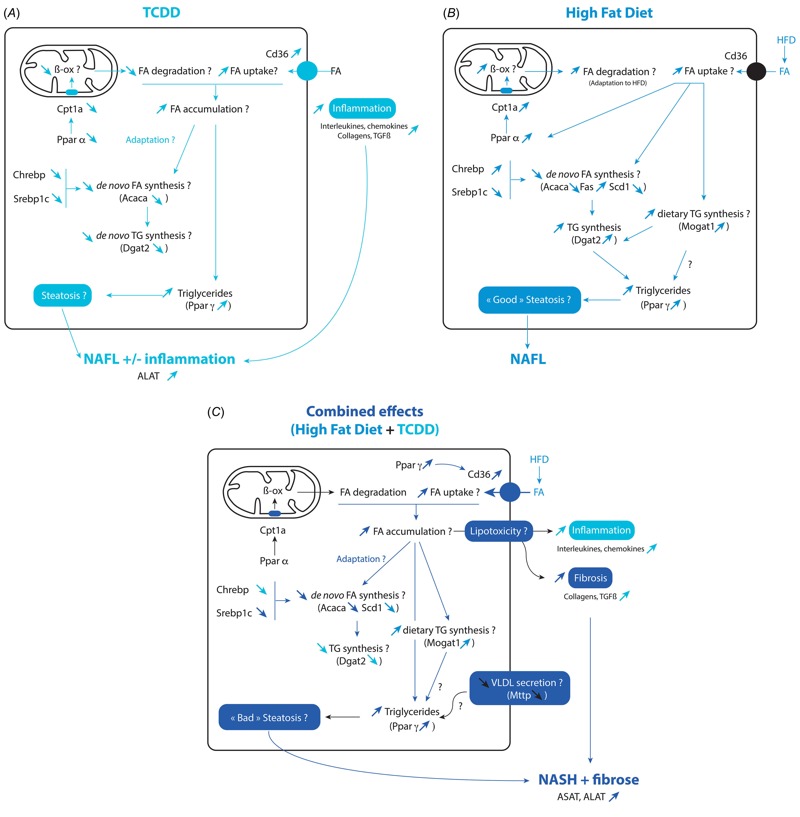
Schema of the effects of TCDD, high fat diet and their combination on liver gene expression and various end points. The effects of TCDD are shown in *A*, for the high-fat diet in *B,* and for their combination in *C* as compared to untreated mice (LF-ctrl). Question marks indicate end points for which the possible modifications have not been measured in the present study.

The effects of TCDD, HFD, and their combination on multiple end points of interest are presented in graphic form in Figure 4. Overall, as compared to LF-ctrl mice, our study suggests that the combined effects of TCDD and HFD are *a*) to increase the uptake of FA (*Cd36*), *b*) to normalize FA β-oxidation (*Cpt1a*), *c*) to decrease *de novo* lipogenesis (*Acaca, Fasn, Scd1*) and carbohydrate metabolism (*Glut2*, *Pklr*), *d*) to decrease *de novo* triglyceride synthesis (*Dgat2/1*) while increasing the monoacylglycerol pathway (*Mogat1*) and *e*) to decrease very low-density lipoprotein secretion (*Mttp*). As previously described, the accumulation of triglycerides in the liver might protect against further hepatic damage and the interruption of triglyceride synthesis is proposed as an initiating event for free FA-mediated lipotoxicity that leads to fibrosis (Choi and Diehl 2008; Trauner et al. 2010).

Importantly, HFD combined with exposure to TCDD led to liver collagen accumulation and increased transaminase levels whereas each treatment alone did not lead to robust changes of these parameters. Consistent with the histological observations, TCDD induced the levels of mRNA of genes that are markers for inflammation, as previously reported (He et al. 2013; Pierre et al. 2014), although there was no further effect with HFD, probably due to the shorter length of our protocol, as opposed to the results of another study (Duval et al. 2010). In contrast, although collagen staining was increased in the co-treated mice (2.6-fold increase) compared to each treatment alone (1.4-fold or 1.2-fold increases for the HF-ctrl or the LF-tcdd groups respectively), the mRNA markers of fibrosis that were tested here were not differentially regulated in the LF-tcdd and HF-tcdd groups. This suggests that other molecular targets underlie the accumulation of collagen such as the regulation of extracellular matrix degradation. For example, both AhR and PPARg modify the expression of the matrix metalloproteases MMP2 and MMP9 that play roles in the development of fibrosis (Pierre et al. 2014; Duval et al. 2002).

In summary, the HFD could lead to an increase of TG synthesis but also to an adaptative increase of fatty acid oxidation. This could limit the steatosis as compared to TCDD that would otherwise lead to a decrease of fatty acid oxidation and TG synthesis but also to an increase of fatty acid uptake. We believe that the combined effects of TCDD and the HFD reflect, mostly, the effects of TCDD when large amounts of fat (through the diet) lead to accumulation of TG in the liver. In addition, TCDD, per se, increases liver inflammation, which, together with the disruption of lipid metabolism that may lead to lipotoxicity, might contribute to the occurrence of fibrosis when combined with HFD. The HFD also might potentiate the effects of TCDD. The HFD might increase the availability of endogenous ligands of AhR, such as tryptophan and its derivatives (Denison and Nagy 2003) that could contribute to the development of obesity-related NAFLD in mice (Moyer et al. 2016). The affinity of AhR for endogenous ligands might be lower than the affinity of exogenous ligands such as TCDD but these endogenous ligands might contribute, nevertheless, to the occurence of TG accumulation with the HFD.

Other studies have reported on the relationship between pollutants, liver abnormalities and diet in mice. Acute exposure to a high dose of TCDD was found to sensitize mice to the development of a NASH with fibrosis following a methionine- and choline-deficient diet (He et al. 2013). The effects of other potential AhR ligands (or mixtures containing AhR ligands) such as polychlorinated biphenyls (Wahlang et al. 2013, 2014; Shan et al. 2015), cigarette smoke (Mallat and Lotersztajn 2009), and diesel particles (Arciello et al. 2013) have been tested on different mouse models of obesity. All these studies suggest that pollutants could be co-factors in the progression of NAFLD in mice. Our study reveals unique features that concern the regulation of gene expression and the development of fibrosis in the livers of obese mice exposed to TCDD.

## Conclusions

We believe that our study helps to unravel the effects of pollutants (TCDD and other AhR ligands) following subchronic exposure in obese mice. It furthers our understanding of the molecular mechanisms that underlie the impairment of liver functions and the development of the final steps of chronic liver diseases, which is crucial for developing preventive measures.

## Supplemental Material

(956 KB) PDF
