# Supplementary material for: Chronic Exposure to Low Doses of Dioxin Promotes Liver Fibrosis Development in the C57BL/6J Diet-Induced Obesity Mouse Model
Source: Environ Health Perspect. 2016 Oct 7;125(3):428–36. doi: 10.1289/EHP316 (PMC5332187; doi:10.1289/EHP316)
Supplement: (956 KB) PDF [file EHP316.s001.acco.pdf]

Environ Health Perspect
DOI: 10.1289/EHP316

Note to readers with disabilities: *EHP* strives to ensure that all journal content is accessible to all readers. However, some figures and Supplemental Material published in *EHP* articles may not conform to 508 standards due to the complexity of the information being presented. If you need assistance accessing journal content, please contact ehponline@niehs.nih.gov. Our staff will work with you to assess and meet your accessibility needs within 3 working days.

Supplemental Material

Chronic Exposure to Low Doses of Dioxin Promotes Liver Fibrosis Development in the C57BL6/J Diet-Induced Obesity Mouse Model

Caroline Duval, Fatima Teixeira-Clerc, Alix F Leblanc, Sothea Touch, Claude Emond, Michèle Guerre-Millo, Sophie Lotersztajn, Robert Barouki, Martine Aggerbeck, and Xavier Coumoul

Table of Contents:

Supplemental Tables

Table S1. Body weight (BW) gain and plasma leptin concentrations after 5 weeks of LFD or HFD.

Table S2. Primer sequences**.**

Supplemental Methods

Animal experiments

Physiologically based pharmacokinetic modeling

Supplemental Results

Determination of the threshold dose of TCDD that induces liver fibrosis

Supplemental References

Supplemental Figures

Figure S1. Distribution of potential low and high gainer individuals in the "TCDD" and "control" subgroups at week 5 of the LFD/HFD intervention. After mice received either a LFD or a HFD for 5 weeks (n=30), body weight (BW) gain and plasma leptin levels were determined, as described in the Methods, to randomize the mice into two subgroups for future injections (from week 8) with 5µg/kg of TCDD (LF-tcdd, n=16 and HF-tcdd, n=16, respectively) or the vehicle (LF-ctrl, n=14 and HF-ctrl, n=14, respectively). A. Variability in BW gain and plasma leptin concentrations at week 5 within the four different subgroups of injection. B. Potential low and high "gainer" status of the mice based on body weight gain and leptin level at week 5 (see also Table S1). In the HFD group, a BW gain of 5 g (and over) and a plasma leptin level of 5 ng/mL (and over) (red lines) were chosen to discriminate potential high gainers (zone 1) and low gainers (zone 2). In the LFD group, low and high gainers do not differentiate yet at week 5, based on those parameters (zone 2). However, the mice with barely detectable leptin levels or with a level of 2.5 ng/mL (and over) might be considered as potential low or high gainers, respectively. All the animals were divided equally in the control and TCDD subgroups. As mentioned in the Methods, two animals were excluded from the study at the time of sacrifice (animals 261 and 227), which belonged respectively to the LF-ctrl and LF-tcdd groups. Data are expressed as mean±SEM; a, versus LF-ctrl; b, versus LF-tcdd; $p<0.001$.

Figure S2. Effect of increasing doses of TCDD on hepatic parameters and on the hepatic mRNA levels of selected genes. Mice were injected with different doses of TCDD (0.1, 1, 10, 25 µg/kg) or the vehicle (Ctrl) during 6 weeks. A. The levels of hepatic *Cyp1a1* mRNA were measured by qPCR (see below). B. Liver weight (LW) as a % of body weight (BW) of the mice. C. The hepatic triglyceride (TG) content. D. Alanine aminotransferase (ALAT) activity in the serum. E. Histological stainings of representative liver sections of the various treatments: hematoxylin-eosin staining (top panel), with red and black

arrowheads indicating lipid droplets and infiltrated inflammatory cells, respectively and picro-sirius red staining (bottom panel), with large black arrows pointing to fibrotic scars of collagen I and III (bar = 100 µm). The hepatic mRNA levels of marker genes for (F) lipid metabolism (*Cd36, Pparg, Plin2/Adrp* and *Dgat2*), (G) inflammation (*Cd68*) and (H) fibrosis (*Tgfb1, Acta2* and *Col1a1*) were measured by qPCR. The relative mRNA levels were estimated using the delta-delta Ct method with *Gapdh* as the reference. Mean expression in the ctrl group is set at 100%. Data are expressed as mean±SEM; *, p<0.05; **, p<0.01; ***, p<0.001; as compared to ctrl; $, p<0.05; $$, p<0.01; as compared to TCDD0.1.

Figure S3. Physiologically-based pharmacokinetic modeling of the levels of TCDD in the organs of mice after intra-peritoneal injections of 5 µg/kg TCDD for 6 weeks. A computerized model was used to estimate the levels of TCDD in the organs of mice following injections with 5 µg/kg TCDD. The time is indicated in hours and the TCDD concentration in ppt. The final TCDD concentrations in ppt wet weight are 32,490 ppt for the adipose tissue (blue curve), 56,834 ppt for the liver (green curve) and 66.8 ppt for blood (red curve).

Supplemental Tables

Table S1. Body weight (BW) gain and plasma leptin concentrations after 5 weeks of LFD or HFD.

group (diet)	subgroup (injection)	mice number	BW gain5	Leptin5
LF	ctrl	236	2.2	3.5
		237	3.9	2.1
		238	2.4	2.6
		239	5.6	2.4
		278	3.4	2.4
		280	3.7	1.6
		222	4.8	1.0
		223	4.3	0.6
		224	4.3	1.9
		225	3.8	0.9
		252	2.1	2.0
		254	3.5	2.8
		261	*4.6*	*1.1*
		263	3.1	1.7
		**MEAN**	**3.7**	**1.9**
		**SD**	**1.0**	**0.8**
	tcdd	241	3.8	0.9
		242	4.3	1.3
		243	4.2	0.4
		244	2.6	1.3
		269	3.0	2.4
		272	5.1	1.8
		275	2.6	2.5
		276	1.8	2.3
		226	2.1	1.9
		227	*4.2*	*0.5*
		229	2.2	2.9
		230	3.4	2.2
		264	1.9	0.5
		265	5.2	7.4
		266	5.3	2.2
		268	3.0	2.2
		**MEAN**	**3.4**	**2.0**
		**SD**	**1.2**	**1.6**

group (diet)	subgroup (injection)	mice number	BW gain5	Leptin5
HF	ctrl	221	5.7	5.2
		228	6.0	1.2
		231	4.3	3.1
		232	4.1	4.9
		257	4.3	3.9
		258	6.9	4.6
		259	7	5.5
		260	9.3	4.7
		245	6.3	0.7
		246	5.8	5.6
		247	6.0	0.2
		248	6.2	7.1
		251	5.9	0.5
		256	7.0	1.3
		**MEAN**	**6.1**[a]	**3.5**
		**SD**	**1.3**	**2.3**
	tcdd	233	4.8	9.7
		234	7.4	5.4
		235	6.9	5.8
		240	10.0	5.6
		262	9.4	6.1
		267	4.7	1.2
		270	6.8	4.6
		271	5.0	0.8
		253	8.7	14.8
		255	6.8	0.5
		273	4.2	2.1
		274	7.8	9.3
		277	5.7	1.0
		279	8.6	4.0
		249	5.7	2.2
		250	9.3	1.0
		**MEAN**	**7.0**[b]	**4.6**
		**SD**	**1.9**	**4.0**

* *: mice that were excluded from further analyses at the time of sacrifice.
Potential high gainer (orange) or low gainer (green) individuals are indicated, based on the two parameters (BW gain, leptin) in the Table. See also Fig. S1.
a, versus LF-ctrl; b, versus LF-tcdd; $p<0.001$.

Table S2. Primer sequences.

Gene Symbol	Forward Sequence	Reverse Sequence
Acaca	AATGAACGTGCAATCCGATTTG	ACTCCACATTTGCGTAATTGTTG
Acta2/aSma	CCTGGTGTGCGACAATG	TGCTCTGGGCTTCATCC
Ccl2/Mcp1	CCCAATGAGTAGGCTGGAGA	TCTGGACCCATTCCTTCTTG
Cd36	TCCAGCCAATGCCTTTGC	TGGAGATTACTTTTCAGTGCAGAA
Cd68	CCAATTCAGGGTGGAAGAAA	CTCGGGCTCTGATGTAGGTC
Col1a1	TCATCGTGGCTTCTCTG	CGTTGAGTCCGTCTTTG
Col3a1	ACGTAGATGAATTGGGATGCAG	GGGTTGGGGCAGTCTAGTG
Cpt1a	GCATGTCAAGCCAGACGAAGAA	TCAGCGAGTAGCGCATAGTCAT
Cyp1a1	ATGAGTTTGGGGAGGTTACTG	AATGAGGCTGTCTGTGATGTC
Dgat1	TCCGTCCAGGGTGGTAGTG	TGAACAAAGAATCTTGCAGACGA
Dgat2	GCGCTACTTCCGAGACTACTT	GGGCCTTATGCCAGGAAACT
Fasn	TCCTGGGAGGAATGTAAACAGC	CACAAATTCATTCACTGCAGCC
G6pc	CCAAGGGAGGAAGGATG	AGGTGACAGGGAACTGCT
Gapdh	GTGGACCTCATGGCCTACAT	TGTGAGGGAGATGCTCAGTG
Gys2	CCAGCTTGACAAGTTCGACA	ATCAGGCTTCCTCTTCAGCA
Hprt	AAGCCTAAGATGAGCGCAAG	TTACTAGGCAGATGGCCACA
Il1b	GCCACCTTTTGACAGTGATG	TCTCCACAGCCACAATGAG
Itgam/Cd11b	GGGAGGACAAAAACTGCCTCA	ACAACTAGGATCTTCGCAGCAT
Mlxipl/Chrebp	GATCCGACACTCACCCACC	CCCGGCATAGCAACTTGAGG
Mogat1	TCCCGTTGTTCCGAGAATATCT	TGCTCAGCACATGAGACAAAC
Mttp	AGCCAGTGGGCATAGAAAATC	GGTCACTTTACAATCCCCAGAG
Pck1/Pepck	CAGGATCGAAAGCAAGACAGT	AAGTCCTCTTCCGACATCCAG
Pklr	CCGCATCTACATTGACGACG	CCGTGTTCCACTTCGGTCAC
Plin2/Adrp	GCCTCTCAACTGGCTGGTAG	ACAGCAAAAGGGGTCATCTG
Ppara	TATTCGGCTGAAGCTGGTGTAC	CTGGCATTTGTTCCGGTTCT
Pparg	CACAATGCCATCAGGTTTGG	GCTGGTCGATATCACTGGAGATC
Ppia/Cyclophilin A	CAGACGCCACTGTCGCTTT	TGTCTTTGGAACTTTGTCTGCAA
Scd1	TTCTTGCGATACACTCTGGTGC	CGGGATTGAATGTTCTTGTCGT
Slc2a2/Glut2	GCACAGACACCCCACTTAC	TCGTCCAGCAATGATGAG
Srebf1/Srebp1c	GGAGCCATGGATTGCACATT	CCTGTCTCACCCCCAGCATA
Tgfb1	ATTCCTGGCGTTACCTTG	CTGATCCCGTTGATTTCC

Supplemental Methods

Animal experiments

Upon arrival, 25 male C57BL/6J mice (Janvier Laboratories) of 6 weeks of age were randomly divided into 5 groups (n=5) and fed a chow diet (V1535R/M-H, Ssniff GmbH). After an acclimatization period of 2 weeks, the mice were injected intra-peritoneally (200 µL/25 g) once a week with either TCDD (0.1, 1, 10 or 25 µg/kg) (LGC Standards) diluted in corn oil or the vehicle (corn oil, Sigma), during 6 weeks. Five days after the last injection, the food was removed from 8 AM to 2 PM. The mice were then anesthetized with isoflurane and blood was drawn through retro-orbital sinus puncture prior to sacrifice by decapitation. The liver was removed, weighed, and either snap-frozen in liquid nitrogen or, for histology, fixed in buffered formalin and processed for paraffin embedding. Serum and plasma samples were obtained after centrifugation of the blood. All of the samples (ctrl n=4, TCDD0.1 n=5, TCDD1 n=5, TCDD10 n=4, TCDD25 n=3) were stored at -80°C until use.

Mice were housed and treated as described in the Methods.

Physiologically based pharmacokinetic modeling

In the present study, the rodent physiologically based pharmacokinetic model developed by Emond *et al.* in 2004 was used.

Supplemental Results

Determination of the threshold dose of TCDD that induces liver fibrosis

The preliminary objective of the study was to determine the threshold subchronic dose of TCDD which induces liver fibrosis. Male C57BL/6J mice were injected once a week with increasing doses of TCDD ranging from 0.1 to 25 µg/kg or its vehicle during 6 weeks and several hepatic parameters were measured. As a marker of exposure to TCDD, we showed that the induction of liver *Cyp1a1* mRNA increased with increasing doses of TCDD (Figure S2A).

Although there was no significant alteration in body weight (BW) during the course of the experiment (data not shown), chronic exposure to increasing doses of TCDD gradually induced hepatomegaly in the mice. The liver weight (LW), expressed as a percentage of the body weight (Figure S2B), increased significantly at a dose of 1 µg/kg of TCDD and represented more than 6% of body weight in mice injected with the highest doses of TCDD (10 and 25 µg/kg). Similarly, liver triglyceride content was moderately increased at 1 µg/kg TCDD (1.5-fold) with large increases at the two highest doses of TCDD tested (2.7 and 3-fold, respectively, for the 10 and 25 µg/kg doses) (Figure S2C). In addition, the 2 highest doses of TCDD led to an increase in serum alanine (Figure S2D) and aspartate (data not shown) aminotransferase activities, which indicates the presence of hepatic lesions.

Liver sections were stained with hematoxylin-eosin and picro-sirius red to evaluate the histopathological changes induced by TCDD. Consistent with the liver triglyceride content, the injection of increasing doses of TCDD for 6 weeks gradually induced liver steatosis. Mice which received an injection of 1µg/kg displayed negligible steatosis (less than 5% of the hepatocytes exhibited lipid droplets). Marked accumulation of lipids was observed in the mice injected with the highest doses of 10 and 25 µg/kg TCDD (Figure S2E, red arrows). The

administration of the highest doses of TCDD (10 and 25 µg/kg) also was associated with an inflammatory cell infiltration (Figure S2E, black arrowheads) whereas the mice injected with the lowest doses of TCDD (0.1 and 1 µg/kg) did not present any signs of liver inflammation. More importantly, liver staining with picro-sirius red showed that the highest doses of TCDD also induced collagen fiber deposition (Figure S2E, large black arrows) whereas the lowest doses had no effect.

To characterize, at the molecular level, the response to the injection of increasing amounts of TCDD, the levels of mRNA of several genes involved in lipid accumulation as well as those of genes that are markers of inflammation and fibrosis were measured by qPCR. All the genes exhibited dose-dependent effects although the threshold dose differed among the genes (Figure S2F, S2G and S2H). The level of *Cd36* mRNA, a membrane transporter involved in lipid uptake and a direct AhR target gene, was increased in all of the mice injected with TCDD as compared to vehicle-treated mice. In contrast, for *Pparg*, a nuclear receptor regulating lipid storage, *Plin2/Adrp*, a major protein for lipid droplet formation, and *Acta2/aSma*, a typical myofibroblast marker, only the mice injected with 10 and 25 µg/kg TCDD exhibited increased levels of mRNA (Figure S2F and S2H) which is consistent with the histological observations (Figure S2E). In contrast, the mRNA level of *Dgat2*, a protein involved in triglyceride synthesis, associated also with liver damage, significantly decreased with injections of increasing concentrations of TCDD, starting at 1 µg/kg (Figure S2F). Furthermore, the expressions of the macrophage marker genes *Cd68* (Figure S2G) and *Itgam/Cd11b* (data not shown) as well as the major pro-fibrogenic cytokine *Tgfb1* and the typical myofibroblast marker *Col1a1* (Figure S2H) increased with increasing amounts of TCDD injected starting at 1 µg/kg.

Fibrotic scars were observed for all the mice that were injected with 10 and 25 µg/kg TCDD whereas the levels of mRNA of genes that are markers for inflammation and fibrosis were

already mildly induced with 1 µg/kg TCDD, indicating that the threshold TCDD dose for fibrosis is encompassed between 1 and 10 µg/kg. Therefore, we chose the intermediate dose of 5 µg/kg TCDD for the diet experiments.

In parallel, in order to determine the relevance of a weekly injection of TCDD in mice over a period of 6 weeks to human exposure, we used a physiologically based pharmacokinetic model. With this model, the intra-peritoneal injection of 5 µg/kg TCDD is predicted to give a final concentration of TCDD in the serum of mice that is below 70 ppt wet weight, which is coherent with values for highly exposed human populations (Figure S3) (Emond et al. 2005).

Supplemental References

Emond C, Birnbaum LS, DeVito MJ. 2004. Physiologically Based Pharmacokinetic Model for Developmental Exposures to TCDD in the Rat. Toxicol. Sci. 80:115–133; doi:10.1093/toxsci/kfh117.

Emond C, Michalek JE, Birnbaum LS, DeVito MJ. 2005. Comparison of the Use of a Physiologically Based Pharmacokinetic Model and a Classical Pharmacokinetic Model for Dioxin Exposure Assessments. Environ. Health Perspect. 113:1666–1668; doi:10.1289/ehp.8016.

Supplemental Figures

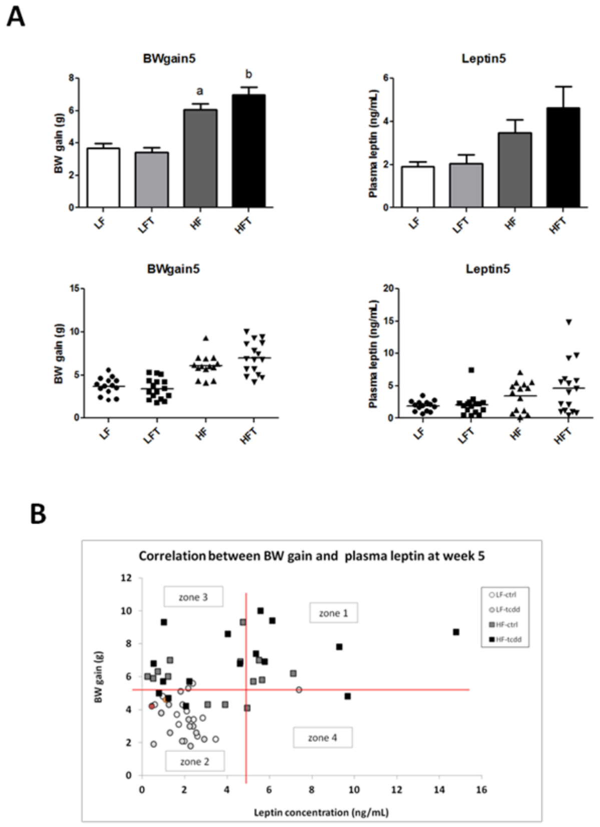

Figure S1. Distribution of potential low and high gainer individuals in the "TCDD" and "control" subgroups at week 5 of the LFD/HFD intervention. After mice received either a LFD or a HFD for 5 weeks (n=30), body weight (BW) gain and plasma leptin levels were determined, as described in the Methods, to randomize the mice into two subgroups for future injections (from week 8) with 5µg/kg of TCDD (LF-tcdd, n=16 and HF-tcdd, n=16, respectively) or the vehicle (LF-ctrl, n=14 and HF-ctrl, n=14, respectively). A. Variability in BW gain and plasma leptin concentrations at week 5 within the four different subgroups of injection. B. Potential low and high “gainer” status of the mice based on body weight gain and leptin level at week 5 (see also Table S1). In the HFD group, a BW gain of 5 g (and over) and a plasma leptin level of 5 ng/mL (and over) (red lines) were chosen to discriminate potential high gainers (zone 1) and low gainers (zone 2). In the LFD group, low and high gainers do not differentiate yet at week 5, based on those parameters (zone 2). However, the mice with barely detectable leptin levels or with a level of 2.5 ng/mL (and over) might be considered as potential low or high gainers, respectively. All the animals were divided equally in the control and

TCDD subgroups. As mentioned in the Methods, two animals were excluded from the study at the time of sacrifice (animals 261 and 227), which belonged respectively to the LF-ctrl and LF-tcdd groups.

Data are expressed as mean±SEM; a, versus LF-ctrl; b, versus LF-tcdd; p<0.001.

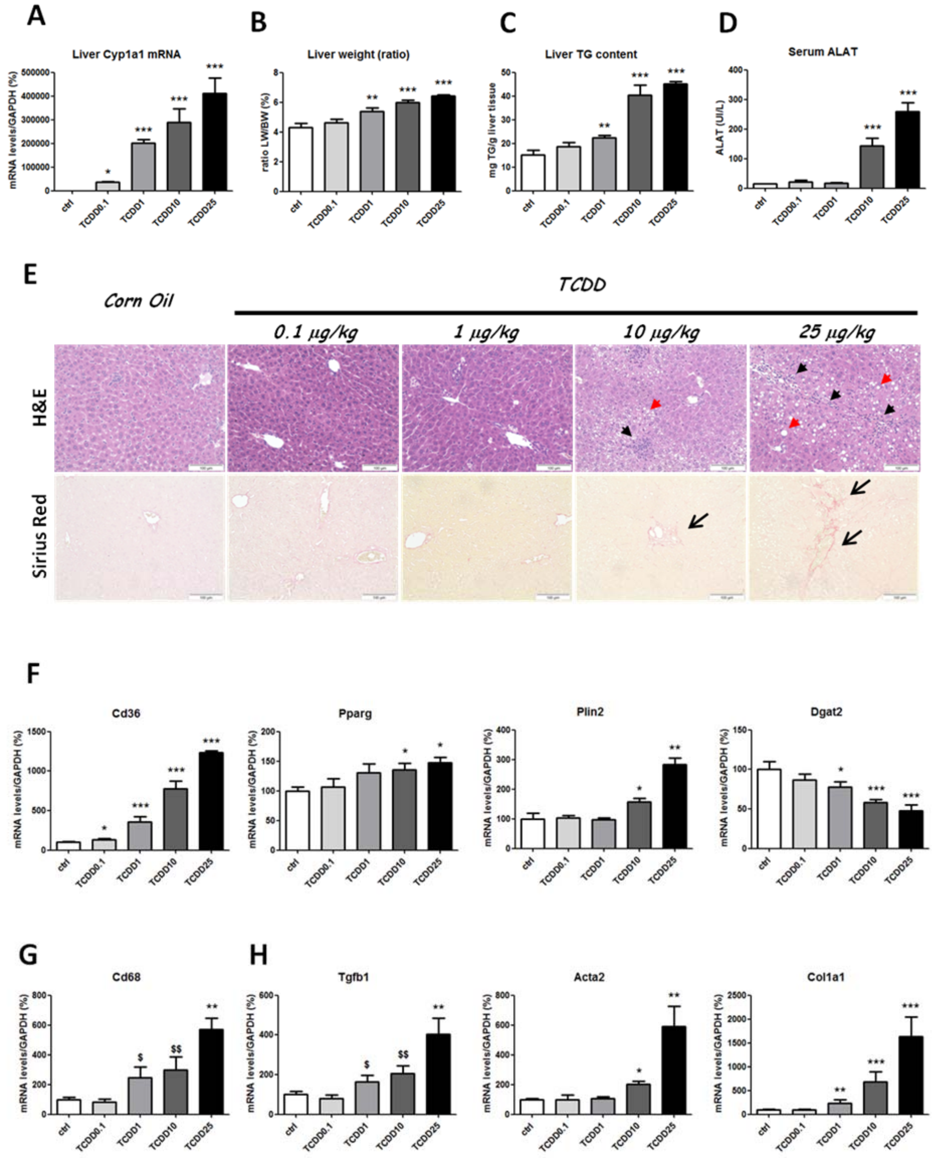

Figure S2. Effect of increasing doses of TCDD on hepatic parameters and on the hepatic mRNA levels of selected genes. Mice were injected with different doses of TCDD (0.1, 1, 10, 25 µg/kg) or the vehicle (Ctrl) during 6 weeks. A. The levels of hepatic *Cyp1a1* mRNA were measured by qPCR (see below). B. Liver weight

(LW) as a % of body weight (BW) of the mice. C. The hepatic triglyceride (TG) content. D. Alanine aminotransferase (ALAT) activity in the serum. E. Histological stainings of representative liver sections of the various treatments: hematoxylin-eosin staining (top panel), with red and black arrowheads indicating lipid droplets and infiltrated inflammatory cells, respectively and picro-sirius red staining (bottom panel), with large black arrows pointing to fibrotic scars of collagen I and III (bar = 100 µm). The hepatic mRNA levels of marker genes for (F) lipid metabolism (*Cd36*, *Pparg*, *Plin2/Adrp* and *Dgat2*), (G) inflammation (*Cd68*) and (H) fibrosis (*Tgfb1*, *Acta2* and *Col1a1*) were measured by qPCR. The relative mRNA levels were estimated using the delta-delta Ct method with *Gapdh* as the reference. Mean expression in the ctrl group is set at 100%.

Data are expressed as mean±SEM; *, p<0.05; **, p<0.01; ***, p<0.001; as compared to ctrl; $, p<0.05; $$, p<0.01; as compared to TCDD0.1.

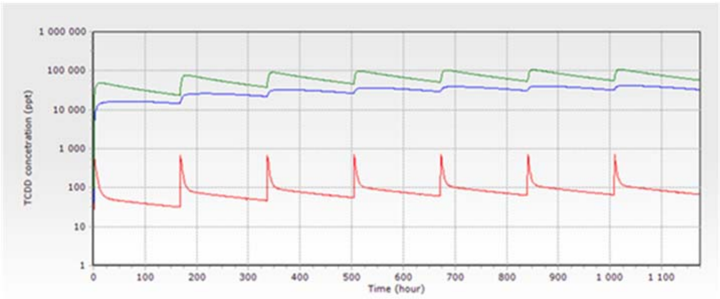

Figure S3. Physiologically-based pharmacokinetic modeling of the levels of TCDD in the organs of mice after intra-peritoneal injections of 5 µg/kg TCDD for 6 weeks. A computerized model was used to estimate the levels of TCDD in the organs of mice following injections with 5 µg/kg TCDD. The time is indicated in hours and the TCDD concentration in ppt. The final TCDD concentrations in ppt wet weight are 32,490 ppt for the adipose tissue (blue curve), 56,834 ppt for the liver (green curve) and 66.8 ppt for blood (red curve).
